# Recent pause in the growth rate of atmospheric CO_2_ due to enhanced terrestrial carbon uptake

**DOI:** 10.1038/ncomms13428

**Published:** 2016-11-08

**Authors:** Trevor F Keenan, I. Colin Prentice, Josep G Canadell, Christopher A Williams, Han Wang, Michael Raupach, G. James Collatz

**Affiliations:** 1 Earth Sciences Division, Lawrence Berkeley National Lab, Berkeley, California 94709, USA; 2 Department of Biological Sciences, Macquarie University, Sydney, New South Wales 2109, Australia; 3 Department of Life Sciences, Imperial College London, Silwood Park Campus, Buckhurst Road, Ascot SL5 7PY, UK; 4 Global Carbon Project, CSIRO Oceans and Atmosphere, Canberra, Australian Capital Territory 2601, Australia; 5 Department of Biology, Graduate School of Geography, Clark University, Worcester, Massachusetts 01610, USA; 6 State Key Laboratory of Soil Erosion and Dryland Farming on the Loess Plateau, College of Forestry, Northwest A & F University, Yangling 712100, China; 7 Biospheric Sciences Laboratory, NASA Goddard Space Flight Center, Greenbelt, Maryland 20771, USA

## Abstract

Terrestrial ecosystems play a significant role in the global carbon cycle and offset a large fraction of anthropogenic CO_2_ emissions. The terrestrial carbon sink is increasing, yet the mechanisms responsible for its enhancement, and implications for the growth rate of atmospheric CO_2_, remain unclear. Here using global carbon budget estimates, ground, atmospheric and satellite observations, and multiple global vegetation models, we report a recent pause in the growth rate of atmospheric CO_2_, and a decline in the fraction of anthropogenic emissions that remain in the atmosphere, despite increasing anthropogenic emissions. We attribute the observed decline to increases in the terrestrial sink during the past decade, associated with the effects of rising atmospheric CO_2_ on vegetation and the slowdown in the rate of warming on global respiration. The pause in the atmospheric CO_2_ growth rate provides further evidence of the roles of CO_2_ fertilization and warming-induced respiration, and highlights the need to protect both existing carbon stocks and regions, where the sink is growing rapidly.

## Abstract (web summary)

Year-to-year variability in atmospheric CO_2_ is strongly influenced by the terrestrial biosphere. Despite increasing anthropogenic emissions, Keenan *et al*. report a recent pause in the growth rate of atmospheric CO_2_ using observations and vegetation models, attributed to an enhanced terrestrial carbon sink.

## Graphical abstract

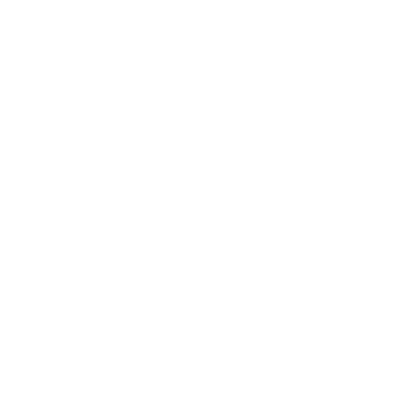

The oceans and the terrestrial biosphere remove about 45% of the CO_2_ emitted by human activities each year^1^. The rate of CO_2_ uptake is not constant, however, and varies greatly from year to year in response to changes in the atmosphere (for example, El Niño events, volcanic eruptions and natural climate variability). The largest component of the year-to-year variability in CO_2_ uptake is contributed by processes on land^2^,^3^. Any increase or decrease in terrestrial uptake thus generates a feedback to the atmosphere^4^, which affects the growth rate of atmospheric CO_2_, and the rate at which the climate warms.

Over the past 50 years, the amount of CO_2_ absorbed by the oceans and terrestrial biosphere annually has more than doubled^1^,^5^,^6^,^7^,^8^. The enhanced carbon sink has been attributed to increased ocean^9^ and terrestrial uptake^1^,^6^,^7^,^8^,^10^, and has occurred despite an increase in the severity and intensity of regional disruptions to ecosystems in recent years, such as extensive droughts, wildfires and insect damage^11^,^12^,^13^,^14^. On land, reports suggest a decline in the tropical sink^15^,^16^, increased plant mortality^17^,^18^ and decreased plant productivity due to droughts and extreme events^19^,^20^. In contrast, others report that elevated CO_2_ has led to increased photosynthesis^8^ and a greening of the biosphere^21^,^22^. The relative contributions of the different processes involved in the terrestrial sink enhancement remain unquantified. Global warming over vegetated land notably slowed since the start of the twenty-first century^23^, while atmospheric CO_2_ concentrations continue to rise, providing an opportunity to test the relative roles of various processes in the enhancement of terrestrial carbon uptake, and examine the implications of enhanced carbon uptake for the growth rate of atmospheric CO_2_.

Here we use extensive ground observations of earth–atmosphere CO_2_ exchange, atmospheric CO_2_ observations and satellite observations of vegetation, along with an ensemble of 10 prognostic dynamic global vegetation models (DGVMs), and a diagnostic process-based modelling approach, to examine the causes of the long-term enhancement of terrestrial carbon uptake and consequences for the growth rate of atmospheric CO_2_. Our analysis suggests that enhanced carbon uptake is due to the combined effects of rising CO_2_ on photosynthesis (the CO_2_ fertilization effect) and, in the past decade, a slowdown in the rate of warming on global respiration. The continued enhancement of the terrestrial carbon sink during the recent slowdown in global warming led to a pause in the atmospheric CO_2_ growth rate, and a decline in the fraction of anthropogenic emissions that remains in the atmosphere.

## Results

### Slowing of the growth rate of atmospheric CO_2_

**Figure 1:**
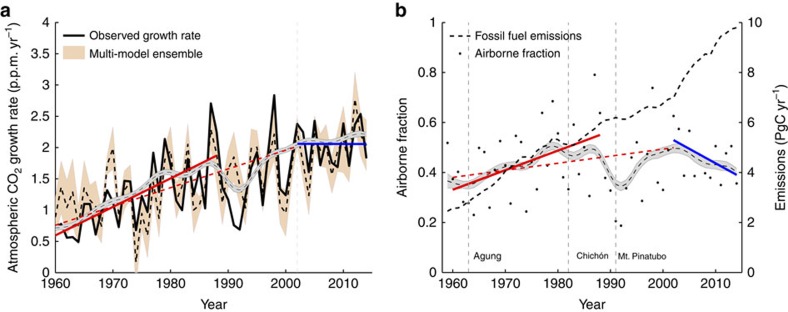
Changes in the airborne fraction and the CO_2_ growth rate. (**a**) Observed (solid black line) and modelled (DGVM ensemble—mean (dashed black line) and s.d. (orange area)) changes in the atmospheric CO_2_ growth rate from 1960 to 2012. The vertical grey line (2002) indicates the point of structural change identified using a linear modelling analysis. The red lines indicate a significant increasing trend from 1959 to 1990 (solid red) and 1959 to 2002 (dashed red) (*P*<0.1), with no trend evident between 2002 and 2014 (blue). All trends are estimated using the non-parametric Mann–Kendall Tau trend test with Sen’s method. The grey area represents the underlying 5-year dynamic (mean±1 s.d.), estimated using SSA. (**b**) Fossil fuel emissions (black dashed line) and the fraction of CO_2_ emissions, which remain in the atmosphere each year (black dots, airborne fraction). Lines indicate significant long-term trends over the periods 1959–1988 (red, increasing) and 2002–2014 (blue, decreasing) at *P*<0.1. The red dashed line shows a slight increasing trend between 1959 and 2002 (*P*=0.18). The grey area represents the underlying 5-year dynamic (mean±1 s.d.), estimated using singular spectrum analysis.

Changes in the residual terrestrial carbon sink affect the proportion of anthropogenic emissions that remain in the atmosphere (the airborne fraction), and thus the growth rate of atmospheric CO_2_. Our analysis suggests that the airborne fraction increased steadily from the 1960s to the 1990s (1.8% per year, *P*=0.03; Fig. 1b), albeit with large interannual variability reflecting year-to-year variability in the terrestrial sink^4^. Since the start of the twenty-first century, however, the airborne fraction has been declining (−2.2% per year, *P*=0.07; Fig. 1b), despite the rapid increase in anthropogenic emissions (Fig. 1b). Changes in the airborne fraction are reflected in the atmospheric CO_2_ growth rate. For the three decades from the start of the measurement record in 1959, the atmospheric CO_2_ growth rate increased from 0.75 to 1.86 p.p.m. per year (Fig. 1a). However, for the period 2002–2014 there has been no significant increase in the growth rate of CO_2_ (Fig. 1a and Supplementary Fig. 1). The decline in the airborne fraction since the start of the twenty-first century has therefore been sufficiently large as to result in a pause in the rate of increase of the atmospheric CO_2_ growth rate (Fig. 1a). Atmospheric growth rates have deviated significantly from predictions of a linear model of atmospheric CO_2_ concentrations and anthropogenic emissions since 2002 (Supplementary Fig. 1), suggesting a nonlinear increase in the global sink strength.

### Enhancement of the terrestrial carbon sink

**Figure 2:**
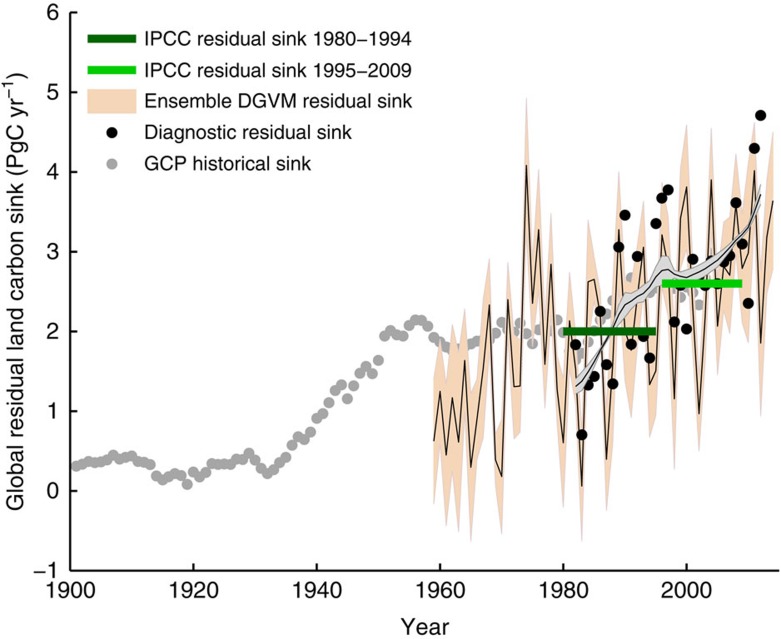
Long-term changes in terrestrial carbon cycling. Estimates of the global terrestrial residual carbon sink from 1901 to 2014. Light grey dots are the historical net residual land CO_2_ sink, estimated by the Global Carbon Project (GCP). Orange shaded areas represent the Global Carbon Project dynamic global vegetation ensemble (annual mean, solid black line, and s.d., orange area) from 10 dynamic global vegetation models (DGVMs). Black dots represent annual values from the satellite-driven diagnostic land surface model, and the grey area represents the associated long-term temporal dynamics (mean±1 s.d.) estimated using singular spectrum analysis. Horizontal bars represent the mean residual land sink values reported by the Intergovernmental Panel on Climate Change (IPCC) 2013, for the periods 1980–1995 (dark green) and 1995–2009 (light green).

Global simulations, from the ensemble of DGVMs included in the Global Carbon Project^1^, and a satellite-based estimate of the terrestrial carbon cycle (see Methods), suggest that the net residual terrestrial carbon sink (the total annual accumulation of carbon in the terrestrial biosphere after accounting for the net effect of land use change) has steadily increased over recent decades, from about 1–2 PgC per year in the 1950s to 2–4 PgC per year in the 2000s (Fig. 2). These model and satellite-based estimates are consistent with recent decadal estimates of the residual terrestrial carbon sink compiled by the Intergovernmental Panel on Climate Change, which show terrestrial uptake increasing from roughly 1.5 PgC per year during the 1980s to 2.6 PgC per year in the 2000s (ref. 24) (Fig. 2), and estimates from the Global Carbon Project (Supplementary Fig. 2).

**Figure 3:**
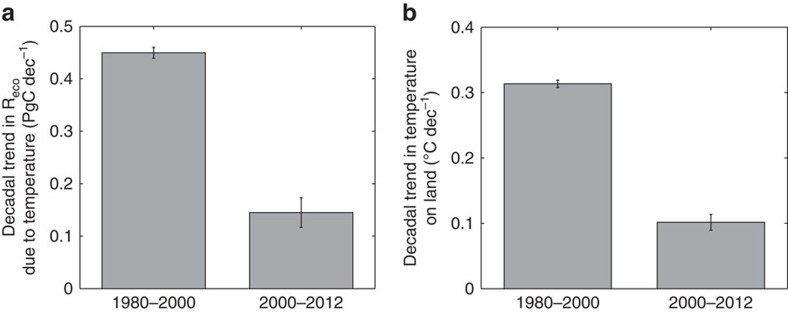
Changes in warming over the land surface and the effect on global ecosystem respiration. Trends in (**a**) ecosystem respiration (R_eco_) derived from satellite-driven estimates of the carbon cycle (photosynthesis-respiration (PR) model, see methods), and (**b**) global warming over vegetated land for the periods of 1980–2000 and 2000–2012. Trends for both periods were estimated using the Sen slope from Kendall's Tau-b method on de-seasonalized monthly data. Error bars represent 95% confidence intervals of the trend.

The slowing of the growth rate of atmospheric CO_2_ between 2002 and 2014 (Fig. 1a and Supplementary Fig. 1) coincides with a period during which global temperature increases over vegetated land also slowed markedly^23^ (Fig. 3 and Supplementary Fig. 3, note recent reports suggest continued warming over oceans^25^). Since the start of the century, global temperatures over vegetated land increased at a rate of 0.1 °C per decade, compared with a rate of 0.32 °C per decade in the previous two decades (Fig. 3). Satellite-driven estimates of the carbon cycle suggest that the slowdown in global warming led to a slowdown in temperature-driven ecosystem respiration of roughly 60% (Fig. 3). The global slowdown in warming exhibited specific regional differences (Supplementary Fig. 4). In particular, although a slowdown in warming was evident over the western United States, and much of Asia, other regions such as the eastern United States, eastern Europe and Siberia experienced accelerated warming (Supplementary Fig. 4). The photosynthesis–respiration (PR) model results suggest that, on a global scale, the lower temperature-driven increase in *R*_eco_ in the past decade, and the continued stimulation of global gross primary production (GPP, see below), likely combined to generate the reported large enhancement of global net ecosystem production (NEP).

### Effects of CO_2_ on photosynthesis and respiration

**Figure 4:**
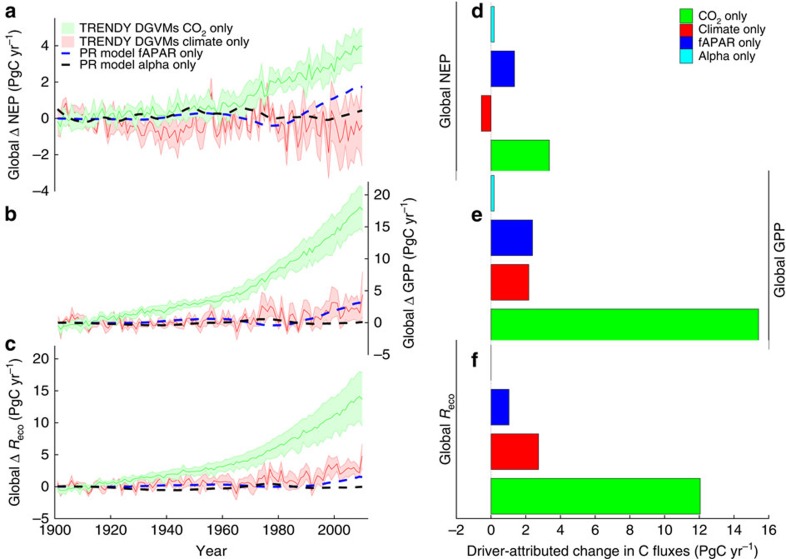
Contribution of different forcings to the long-term change in terrestrial carbon cycling. Model estimates of the extent to which long-term changes in different forcing factors are responsible for the long-term change in net ecosystem production (NEP) (**a**,**d**), gross primary production (GPP) (**b**,**e**) and ecosystem respiration (*R*_eco_; **c**,**f**), where NEP=GPP−*R*_eco_. Shaded areas in **a**–**c** represent the mean and s.d. from the TRENDY ensemble of dynamic global vegetation model (DGVM) simulations with varying CO_2_ (green) or climate (red) only. Dashed lines in **a**–**c** show the effect of changes in vegetation (or the fraction of absorbed radiation (fAPAR), blue), and water availability (Alpha, black) estimated using a satellite-driven coupled photosynthesis-respiration (PR) model. (**d**–**f**) The mean change associated with each driver between the periods 1901–1915 and 1995–2010.

**Figure 5:**
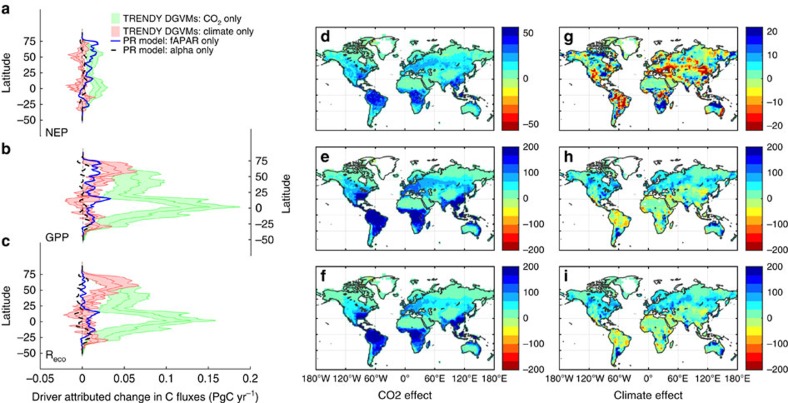
Global distribution of change. (**a**–**c**) The latitudinal distribution of the effect of changes in different forcing factors on (**a**) net ecosystem production (NEP), (**b**) gross primary production (GPP) and (**c**) ecosystem respiration (*R*_eco_). Shaded areas represent the mean and s.d. from the TRENDY ensemble of dynamic global vegetation models (DGVM) simulations with varying only CO_2_ (green) or climate (red). Dashed lines in **a**–**c** show the effect of changes in vegetation (or the fraction of absorbed radiation (fAPAR), blue) and water availability (Alpha, black) estimated using a diagnostic coupled photosynthesis-respiration (PR) model. (**d**–**i**) The spatial distribution of the influence of increasing atmospheric CO_2_ and changes in global climate on total rates of NEP (**d**,**g**), GPP (**e**,**h**) and total ecosystem respiration (**f**,**i**) in gC m^−2^ per year. Effects are estimated based on the difference between the 15-year periods of 1901–1915 and 1995–2010.

Although a lack of temperature increases likely contributed to the slowdown in the growth rate of atmospheric CO_2_ over the past decade (Fig. 3), results from the DGVM ensemble suggest that an increasing atmospheric CO_2_ concentration was the primary driver of the enhanced uptake over the past century (Fig. 4). Atmospheric CO_2_ concentrations increased from roughly 290 p.p.m. at the start of the twentieth century to 400 p.p.m. by 2015, with a pronounced effect on global GPP (Fig. 4b), and a large but lesser effect on *R*_eco_ (Fig. 4c). The DGVM simulations suggest that increasing atmospheric CO_2_ concentrations led to an increase in global annual GPP of 18±2 PgC (mean±1 s.d.) since 1900 (Fig. 4b). Elevated CO_2_ also increased *R*_eco_ due to the carbon supplied through photosynthesis (Fig. 4c). Empirical evidence for this link has been reported across a range of ecosystems: grasslands^26^; crops^27^; and forests^28^. The DGVM results suggest an increase of 13±4 PgC in global annual *R*_eco_ over the past century due to increasing CO_2_ (Fig. 4c). The largest increases in absolute terms were located in the tropics for both GPP and *R*_eco_ (Fig. 5), with a lesser contribution from northern temperate and boreal regions.

### Effects of changes in vegetation cover and climate

The direct effects of changes in atmospheric CO_2_ on both photosynthesis and respiration have had a larger impact on the terrestrial carbon sink than changes in climate, vegetation cover and water availability (Fig. 4). The effects of changes in climate are particularly evident in the 1980s and 1990s, when temperature increases were most pronounced (Fig. 3), with the largest impacts at higher latitudes (Fig. 5). Overall, the impact of changes in climate on global net carbon uptake is uncertain, due to the small magnitude of the effect compared with differences between the DGVM models. It is worth noting however that there is a strong indirect effect of temperature through the alleviation of temperature limitations to growth in colder regions, and the extension of the growing season, observable as an increase in vegetation cover in satellite observations.

A global greening of the Earth’s surface has been reported in satellite observations^21^,^22^, which has been attributed to both the direct effect of temperature changes and the indirect effect of CO_2_ fertilization^21^,^22^. The results from our satellite-based carbon cycle model suggest that this has increased the residual terrestrial sink by about 0.66 PgC per decade over the past three decades (Fig. 4). The largest effects are evident at northern latitudes, where warming over the past century has diminished temperature limitations (Fig. 5). The effect of greening on the global carbon cycle was secondary to the direct effect of increasing atmospheric CO_2_ on GPP and *R*_eco_, and that of increasing temperatures on *R*_eco_ (Fig. 4).

Drought has been suggested to increase under future warming^11^,^29^, due to the effect of higher global temperatures on potential evapotranspiration rates, leading to an expected decrease in green vegetation in some regions^30^. We do not detect a change in soil moisture availability using global climate data (Fig. 4), and attribute only a small fraction of the long-term change in global carbon uptake to changes in the water cycle (Fig. 5). The lack of a global change in soil moisture availability is in line with recent reports^14^,^31^ (but see ref. 29). Although global warming is often associated with an increase in the prevalence of drought, global precipitation increases with global temperatures^32^. Collectively, this suggests that there has been little to no change in the prevalence of drought over recent decades on a global scale^14^,^31^ despite the occurrence of large regional drought events (for example, ref. 33).

## Discussion

The ongoing enhancement of CO_2_ uptake by the terrestrial biosphere is slowing the rate atmospheric CO_2_ accumulation. Both theory and observations suggest CO_2_ fertilization as a likely, dominant explanation of the global enhancement^8^,^34^, though alternative perspectives exist^35^. Our results suggest that the direct effect of CO_2_ on both photosynthesis and respiration is much larger than the indirect effect of global land surface greening^21^ and global changes in soil moisture^18^. In the most recent decade, results suggest that terrestrial uptake has increased as a consequence of a slowdown in the rate of global warming over vegetated land, resulting in a decline in the rate of increase in global respiration. We show that the combined effect of CO_2_ fertilization and the slowdown in warming has been sufficiently large to decrease the airborne fraction of anthropogenic CO_2_ emissions and slow the growth rate of atmospheric CO_2_ despite increasing anthropogenic emissions. Model simulations predicted largest changes to the terrestrial carbon sink in both tropical ecosystems, due to the effect of CO_2_ on photosynthesis, and high-latitude ecosystems, due to land surface greening and the effects of both CO_2_ and temperature on photosynthesis and respiration. It is important to note that the land sink is due to the lag between carbon uptake through photosynthesis and release through respiration. Although the sensitivity of GPP to rising CO_2_ is expected to decline as CO_2_ concentrations rise (see Methods), the observed enhancement will thus likely persist into the future as long as the stimulation of productivity by elevated CO_2_ continues to outweigh net carbon releases from warming. The slowdown in global warming is expected to be temporary^23^ however and may already have ended with the strong El Niño Southern Oscillation of 2015 and 2016, with subsequent consequences for the growth rate of atmospheric CO_2_ (ref. 36). The likely continuation of warming in the coming decades^37^ suggests further future increases in net carbon releases.

Other factors not examined here could contribute to changes in the residual terrestrial sink, including nutrient deposition^38^, changes in diffuse light^39^ and ozone concentrations^40^. Nutrient deposition has been reported to increase forest growth, particularly in areas of high N-deposition, primarily north-eastern United States, Western Europe and north-eastern China. Recent studies have estimated the effect of N-deposition on global forest growth on the order of 0.3% per year^38^. Much less is known about the effect of N-deposition on decomposition processes, however, with recent reports suggesting that N-deposition inhibits respiration from forest soils^41^. Modelling studies^42^,^43^ indicate an increase in global annual terrestrial uptake of about 0.2–0.24 PgC in recent decades due to anthropogenic N-deposition, which is an order of magnitude lower than our estimates of recent changes in carbon uptake due to observed changes in fraction of absorbed photosynthetically active radiation (fAPAR) alone (Fig. 4). Diffuse light changes have also been reported and are estimated to have increased uptake between 1960 and 1980 by 0.44 PgC per year globally^39^. The effect of ozone on the terrestrial carbon cycle is uncertain, due to unknowns regarding plant-specific sensitivities to ozone^40^ and the effect of canopy structure^44^. Ultimately, there are a myriad of factors that influence the carbon cycle, particularly at regional scales. Our analysis suggests however that CO_2_ and temperature are most likely the dominant factors driving global long-term change.

Despite the decline in the airborne fraction and the resulting pause in the growth rate of atmospheric CO_2_, the ultimate outcome regarding the pace and magnitude of climate change depends heavily on future emission pathways. CO_2_ emissions, through the burning of fossil fuels, cement production and land use, have continued to track close to the high end of all scenario predictions^24^. Enhanced carbon uptake by the biosphere to date has served to slow the growth rate of atmospheric CO_2_ and our results support the hypothesis that net terrestrial CO_2_ uptake has been especially strong recently^45^. Without effective reduction of global CO_2_ emissions, however, future climate change remains a stark reality.

## Methods

### Global carbon cycle data

We used global carbon budget data from the Global Carbon Project^1^, in combination with diverse observational data sets from satellite remote sensing and distributed Earth observation networks, multiple prognostic DGVMs and a diagnostic modelling approach. The Global Carbon Project^1^ data set is a compilation of estimates of all major components of the global carbon budget, based on the combination of a range of data, algorithms, statistics and model estimates. This data set provided the long-term estimates of global emissions, along with estimates of the residual terrestrial sink from 10 DGVMs. Long-term atmospheric CO_2_ concentrations were provided by the National Oceanic and Atmospheric Administration’s (NOAA) Earth System Research Laboratory ( http://www.esrl.noaa.gov). Annual emissions of CO_2_ from the burning of fossil fuels, land use change and cement production from the Global Carbon Project^1^ were used in conjunction with the NOAA atmospheric CO_2_ concentration data to calculate the annual airborne fraction.

### Diagnosing changes in the growth rate of atmospheric CO_2_

We used two methods to examine changes in the growth rate of atmospheric CO_2_ over time: a statistical linear model of the growth rate as a function of emissions, atmospheric CO_2_ concentrations and global CO_2_ sinks^46^. Rayner *et al*.^46^ showed that the growth rate can be modelled as a linear function of atmospheric CO_2_ concentration, and that deviations of the atmospheric growth rate from this linear model are an indication of changes in the sink strength of the biosphere. This is an informative approach as it incorporates the physical link between atmospheric CO_2_ concentrations and global CO_2_ sinks, thus allowing changes in that coupling to be studied. By examining the residuals of this linear model we identify a significant residual bias from 2002 to 2014, indicating a structural change in the relationship between atmospheric CO_2_ concentrations, emissions and global CO_2_ sinks (Supplementary Fig. 1). The second method, singular spectrum analysis (SSA), is used to extract the underlying temporal dynamics of both the growth rate and the airborne fraction.

### Examining changes in atmospheric CO_2_ with linear modelling

The growth rate can be modelled as a linear function of atmospheric CO_2_ concentrations^46^. Deviations of the atmospheric growth rate from this linear model are an indication of changes in the sink strength of the biosphere. Such an approach is more informative than a simple linear model as a function of time, as it incorporates the physical link between atmospheric CO_2_ concentrations and global CO_2_ sinks, thus allowing changes in that coupling to be studied.

Formally, the global CO_2_ sink can be described as a linear function of CO_2_ concentration or, equivalently, CO_2_ mass. Thus, we write


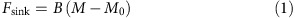


where *M* is the mass of CO_2_ in the atmosphere and *M*_0_ is the background or equilibrium mass of CO_2_ in the atmosphere. Equation (1) can be simplified to


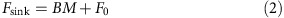


where *B* has units of per year and plays the role of an inverse residence time for excess carbon against the processes of land and ocean uptake.

Given that


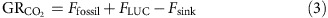


where 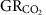 is the growth rate of atmospheric CO_2_. We can substitute equation (2) into equation (3) to get


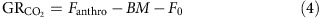


Using observations of emissions from fossil fuel burning and land use change (*F*_anthro_), along with the atmospheric CO_2_ concentrations and growth rate, it is possible to estimate *B* and *F*_0_ using standard statistical techniques.

The model thus constructed preserves the relationship between atmospheric CO_2_ concentrations, emissions and the growth rate of atmospheric CO_2_, on the basis of a proportional response of the global CO_2_ sinks. Any change in the strength of the global sinks can therefore be analysed by examining the residuals between the observed and predicted growth rate. We use the model, informed by the first 30 years of observations to test the hypothesis that the CO_2_ growth rate maintains the same linear relationship with atmospheric CO_2_ concentrations throughout. The residuals show a statistically significant deviation from being normally distributed around zero from 2002 (*P*<0.05, *t*-test, Supplementary Fig. 1). This shows that there is a pause in the growth rate of atmospheric CO_2_. Importantly, it also identifies changes in global CO_2_ sinks as the cause. We confirm this change in the growth rate by using SSA to extract the low frequency mode of variability corresponding to 5 years (see below).

### Examining changes in atmospheric CO_2_ with SSA

SSA is a non-parametric spectral estimation method for extracting different modes of variability form a time series. The SSA method decomposes time series into a sum of components, each having a meaningful interpretation. Different modes of underlying variability can then be extracted by reconstructing the time series using only the eigenvalues relevant to the mode of variability in question. The name singular spectrum relates to the spectrum of eigenvalues in a singular value decomposition of a covariance matrix.

SSA first decomposes the time series into a set of empirical orthogonal functions and associated principal components in the spectral domain, following Takens’ embedding theorem^47^. Each component is dominated by a single oscillatory mode, and therefore has a simple representation in the frequency domain. This means that each empirical orthogonal function can thus be assigned a characteristic frequency. The functional separation from the decomposition step can be used to reconstruct the time series, either fully or only retaining specific modes of variability by using the relevant principal components. Thus, a time series can be finally described by a set of subsignals, *X*_f_, each of which belongs to a well-defined frequency bin. All SSA analyses in this paper were performed using a 5-year frequency.

Despite of orthogonal base functions, extracted subsignals are subject to uncertainty due to a degree of inseparability of closely related modes of variability^48^. To quantify this uncertainty, we use a surrogate technique (the Iterative Amplitude Adjusted Fourier Transform^49^), following Mahecha *et al*.^50^ This approach generates a set of surrogates for each residual, corresponding to the extracted 5-year subsignal. The subsignal *X*_5_ is then re-extracted 100 times, giving an array of subsignal surrogates. The s.d. of the extracted subsignals quantifies the extraction uncertainty, and is shown in Fig. 1.

### DGVMs of the terrestrial carbon cycle

DGVM output from two different model intercomparison projects was used in this analysis (Supplementary Table 1). As part of the Global Carbon Project^1^, transient runs from 10 DGVMs are available from 1959 to 2014 (www.globalcarbonproject.org/carbonbudget). These model simulations were assessed using observed atmospheric CO_2_ growth rates (Supplementary Fig. 2) and used to explore the decadal change in the land sink from 1959 to 2014 (Fig. 2). See Le Quere *et al*.^1^ for a detailed description of the models. We also use global simulations from 8 DGVMs (Supplementary Table 1) run as part of the Trends in Net Land-Atmosphere Exchange (TRENDY-v1) project (http://dgvm.ceh.ac.uk/node/9). In this project, common input forcing data were prescribed for a series of model experiments from 1901 to 2010. Here we use two model experiments from TRENDY-v1, varying either CO_2_ only (S1 (ref. 7): time-invariant climate; present-day land use mask) or climate only (S4 (ref. 51): time-invariant CO_2_; present-day land use mask). For more details on the TRENDY model simulations see Sitch *et al*.^7^

### Satellite-based estimates of the terrestrial carbon cycle

In addition to the DGVMs, we used satellite-based estimates of ecosystem GPP combined with ecosystem respiration estimates from a diagnostic coupled PR model to quantify the likely effect of changes in global vegetation (through the fAPAR), and changes in water availability (though Alpha) on enhanced terrestrial uptake, from 1982 to 2013.

The diagnostic coupled PR model is based on a new light use efficiency (LUE) model of photosynthesis developed from first principles, and a semi-empirical model of ecosystem respiration developed based on eddy-covariance flux data.

The mechanistic photosynthesis model proposed by Farquhar *et al*.^52^ successfully captures the biochemical controls of leaf photosynthesis and responses to variations in temperature, light and CO_2_ concentration. According to the model, the gross photosynthesis rate, *A*, is limited by either the capacity of the Rubisco enzyme for the carboxylation of RuBP (ribulose-1,5-bisphosphate), or by the electron transport capacity for RuBP regeneration.

In the case of Rubisco limitation, the photosynthetic rate (*A*_*c*_) is given by


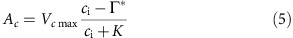


where *V*_*c*max_ is the maximum rate of Rubisco activity, *c*_i_ is the intercellular concentration of CO_2_, Γ* is the CO_2_ compensation point in the absence of dark respiration and *K* is the Michaelis–Menten coefficient of Rubisco.

In the case of limitation by the electron transport capacity for RuBP regeneration, and assuming electron transport capacity is large (relative to *V*_*c*max_) such that the response of photosynthesis to light is linear under Rubisco limitation, the photosynthetic rate (*A*_j_) is given by


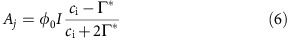


where *φ*_0_ is the intrinsic quantum efficiency and *I* is the absorbed light.

The co-limitation or coordination hypothesis, which is strongly supported by empirical evidence^53^,^54^, predicts that photosynthesis under typical daytime field conditions is close to the point where Rubisco- and electron transport-limited photosynthesis rates are equal (that is, equation (5)=equation (6))^55^. In other words, the photosynthetic capacity of leaves adjusts to acclimate to the typical daytime light levels to be neither in sufficient excess to induce additional, non-productive maintenance respiration nor less than required for full exploitation of the available light. Recent empirical support comes from Maire *et al*.^53^, who tested the coordination hypothesis with 293 observations for 31 species grown under a range of environmental conditions, and found that average daily photosynthesis under field conditions is close to the point, where the Rubisco and electron transport photosynthesis rates are equal.

The coordination hypothesis allows for the prediction of photosynthesis through equation (6) using a LUE approach. Indeed the success of LUE models generally in predicting photosynthesis can be explained by the co-ordination hypothesis. Importantly, it also allows for the effect of CO_2_ on photosynthesis to be incorporated in such LUE models based on the first-principles understanding of the Farquhar *et al*.^52^ model.

By rewriting equation (6), substituting *c*_i_ by the product of atmospheric CO_2_ (*c*_a_) and the ratio of leaf-internal to ambient CO_2_ (*χ*=*c*_i_/*c*_a_), GPP can be described as


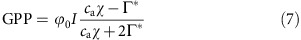


where *φ*_0_ is the quantum yield and *I* is the absorbed light (here derived from satellite fAPAR).

Γ* depends on temperature, as estimated through a biochemical rate parameter (*x*) as described by Bernacchi *et al*.^56^:


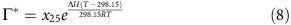


here *R* is the molar gas constant (8.314 J mol^−1^ K^−1^) and *x*_25_=4.22 Pa is the photorespiratory point at 25 °C. Δ*H* is the activation energy for Γ* (37,830 J mol^−1^) and *T* is the temperature in K.

*χ* depends on air temperature and the vapour pressure deficit (VPD; *D*), and can be estimated following the least-cost hypothesis^54^. This states that an optimal long-term effective value of *χ* can be predicted as a result of plants minimizing their total carbon costs associated with photosynthetic carbon gain, and explicitly expressed with the following model


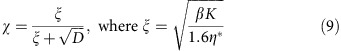


where *D* is VPD, *K* is the Michaelis–Menten coefficient of Rubisco and η* is the viscosity of water relative to its value at 25 °C. *D* is estimated as the difference between saturated and actual vapour pressure. Saturated vapour pressure (*e*_s_) is estimated as the averaged saturated vapour pressure at maximum and minimum temperature with the Clausius–Clapeyron relationship, which is well approximated by


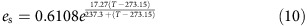


The Michaelis–Menten coefficient of Rubisco (*K*) in equation (9) is given by


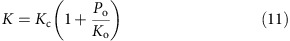


where *K*_c_ and *K*_o_ are the Michaelis–Menten coefficient of Rubisco for carboxylation and oxygenation, respectively, expressed in partial pressure units, and *P*_o_ is the partial pressure of O_2_. *K* responds to temperature via *K*_c_ and *K*_o_, which is also described by equation (8) with specific parameters: Δ*H* is 79.43 kJ mol^−1^ for *K*_c_ and 36.38 kJ mol^−1^ for *K*_o_, *x*_25_ is 39.97 Pa for *K*_c_ and 27,480 Pa for *K*_o_. On the basis of equations (9)–(11), 11, 12, and assuming a typical value of *χ*_25_ as 0.8 (at *T*=298.15 K and *D*=1 kPa), parameter *β* of equation (9) is estimated as 356.51.

The GPP model implicitly assumes that nutrient limitations on GPP are manifest in allocation to foliage and are therefore contained in the observed fAPAR, as has been reported in recent empirical observations^57^,^58^. This is consistent with the theoretical expectation and empirical evidence that CO_2_-induced enhancement of biomass growth is possible even under nutrient-limited conditions^59^, and findings of increased below-ground allocation, including root exudation, on less fertile soils^60^.

Ecosystem respiration (*R*_eco_) in the diagnostic model is estimated via a photosynthesis-dependent respiration model^61^, which combines the joint influences of temperature, soil moisture and substrate availability on ecosystem respiration, and is designed for the diagnostic upscaling of *R*_eco_ from observations at eddy-covariance towers to global scales. The model estimates monthly *R*_eco_ as


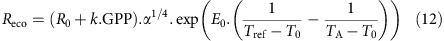


where *R*_0_ is the reference respiration rate at the reference temperature *T*_ref_ (15 °C), *E*_0_ is the activation energy and *T*_0_=−46.02 °C (ref. 61). *k* is the proportional contribution of GPP to ecosystem respiration through substrate availability at the reference temperature *T*_ref_, and is a measure of water availability, calculated as the ratio of actual to equilibrium evapotranspiration (see below). Conceptually, this model can be considered as the sum of a GPP-dependent term comprising autotrophic respiration and the fast-responding labile component of heterotrophic respiration, and a GPP-independent term standing for heterotrophic respiration of slower carbon pools. The two free model parameters (*E*_0_, *k*) were taken from the original study^61^, where 104 globally distributed sites from the FLUXNET network were used to derive plant functional type (PFT) specific parameters. Global PFT classifications were taken from the MODIS land cover product (MOD12, http://modis-land.gsfc.nasa.gov/landcover.html), curated at a resolution of 0.5° by the Global Land Cover Facility (http://glcf.umd.edu/data/lc/). For each 0.5° grid cell, we used the PFT that was most prevalent during the period 2000–2013. *R*_0_ was estimated for each 0.5° grid cell analytically, by solving the combined equations (7) and (12) for equilibrium net carbon uptake under preindustrial conditions.

### Diagnostic model simulations performed

To examine the role of changes in each of the model drivers (air temperature, atmospheric CO_2_, radiation, moisture availability and vegetation cover) used in our analysis, we ran multiple global 0.5° simulations from 1900 to 2013 with the PR model. For each simulation, we removed the long-term trend in all drivers but one. This allowed us to quantify the direct first-order effect of long-term changes in each. An fAPAR climatology was used pre-1981.

### Diagnostic model forcing data

Global monthly gridded weather data at 0.5° were provided by the Climate Research Unit at East Anglia University (CRU TS3.21)^62^. The total available photosynthetically active radiation, VPD and the ratio of actual to equilibrium evapotranspiration (*α*) were calculated from insolation and CRU climate data using a simple process-based bioclimatic model (STASH^63^). The GIMMS_3G_ remotely sensed Normalized Difference Vegetation Index product^64^ provided monthly estimates of the fAPAR, an indicator of green vegetation cover, at 0.5°. Monthly fAPAR estimates are available from 1981 to the present.

### Diagnostic model evaluation

We evaluated the efficacy of the PR model at multiple temporal and spatial scales. Evaluations performed include the following: a global comparison to interannual variability in the residual terrestrial sink from multiple DGVMs and estimates from the Global Carbon Project^65^ (Supplementary Fig. 2), site-level comparisons to time series of GPP and *R*_eco_ from globally distributed individual sites in the La Thuile Fair Use FLUXNET data set (Supplementary Figs 5–8 and Supplementary Table 2); comparisons with the seasonal anomalies of GPP and *R*_eco_ from 149 sites from the same data set (Supplementary Fig. 9); regional comparisons with seasonal changes in atmospheric CO_2_ concentrations from the NOAA global sampling stations (Supplementary Fig. 10); and a latitudinal comparisons to an empirical upscaling estimate of global GPP^66^ (Supplementary Fig. 11).

To compare PR model estimates of NEP to the observations at the NOAA stations, we used the TM2 atmospheric transport model^67^ to integrate and transport detrended monthly values of NEP for each 0.5° grid cell to the station locations. We then calculated both the modelled and observed CO_2_ seasonal cycle at the observation sites^68^.

### Derivation of the sensitivity of GPP to CO_2_

GPP can be described as a function of atmospheric CO_2_ as


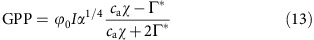


The sensitivity of GPP to *c*_a_ can therefore be derived by taking the derivative of GPP with respect to *c*_a_, as


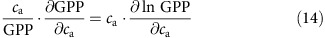


This requires the derivation of 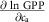, which can be formulated as:


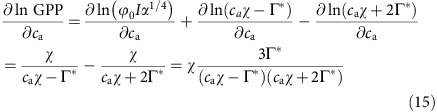


It therefore follows that


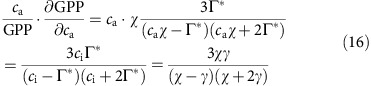


Taking *χ*=0.8 and Γ*=43 p.p.m. (at 25 °C), the sensitivity of GPP is thus calculated as 37% at current levels of atmospheric CO_2_ (400 p.p.m.).

Examining the first derivative of the LUE model of GPP^54^,^58^ suggests a CO_2_ sensitivity (*β*_CO2_) of GPP of 37% at current atmospheric CO_2_ levels (400 p.p.m.), which is consistent with the observed response in FACE studies^69^. Our estimate of the change in GPP is also consistent with other process-based estimates^7^,^8^, but is larger than estimates from commonly used empirical upscaling techniques (Supplementary Fig. 12) as these do not account for the effect of increasing atmospheric CO_2_ on photosynthesis.

### Long-term change in the sensitivity of GPP to changing *c*_a_

The change in the sensitivity of GPP to *c*_a_ can also be derived analytically.

If we denote the sensitivity (from equation (16)) as


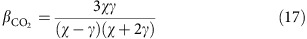


Then we can calculate the partial derivative of *β*_CO2_ as


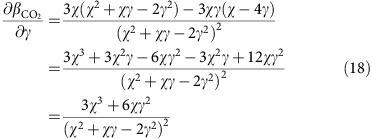


And the partial derivative of gamma as


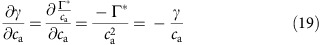


Combining equations (18) and (19) gives


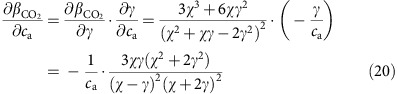


The negative coefficient implies that the sensitivity of GPP to *c*_a_, *β*_CO2_, will decline with increasing *c*_a_. It is also clear from equation (20) that the response of *β*_CO2_ to *c*_a_ is not linear, but decreases with *c*_a_. In other words, the magnitude of the sensitivity declination decreases with *c*_a_ enhancement. Evaluating *β*_CO2_ at different atmospheric CO_2_ concentrations shows a decrease in *β*_CO2_ from 37% under current CO_2_ levels to 19% at double the current CO_2_ levels (Supplementary Table 3).

### Data availability

Data used in this study are available from the Global Carbon Project data archive at the Carbon Dioxide Information Analysis Center (http://cdiac.ornl.gov/GCP/). This includes global carbon budget data and long-term simulations from DGVMs. Additional simulations used are available from the Trends in Net Land-Atmosphere Exchange (TRENDY) project (http://dgvm.ceh.ac.uk/node/9). All other data that support the findings of this study are available from the corresponding author on request.

### Code availability

The code used in this study is available from the corresponding author on request.

## Additional information

**How to cite this article:** Keenan, T. F. *et al*. Recent pause in the growth rate of atmospheric CO_2_ due to enhanced terrestrial carbon uptake. *Nat. Commun.* **7,** 13428 doi: 10.1038/ncomms13428 (2016).

**Publisher's note:** Springer Nature remains neutral with regard to jurisdictional claims in published maps and institutional affiliations.

## Supplementary Material

Supplementary Information Supplementary Figures 1-12 and Supplementary Tables 1-3 Supplementary References.
