# Corrigendum: Recent pause in the growth rate of atmospheric CO_2_ due to enhanced terrestrial carbon uptake

**DOI:** 10.1038/ncomms16137

**Published:** 2017-07-14

**Authors:** Trevor F. Keenan, I. Colin Prentice, Josep G. Canadell, Christopher A. Williams, Han Wang, Michael Raupach, G. James Collatz

Nature Communications 7: Article number:13428; DOI: 10.1038/ncomms13428 (2017); Published 11 08 2016; Updated 07 14 2017

An earlier publication by Leggett and Ball presented statistical evidence for a relationship between the pause in global temperature, a pause in the global rate of change of CO_2_ and an increase in global vegetation cover.

While this publication was initially omitted from the reference list of this Article, the authors acknowledge that given the overlap between the two studies and marked differences in their conclusions, citation of this earlier work is appropriate.

Leggett, L. M. W. & Ball, D. A. Granger causality from changes in level of atmospheric CO_2_ to global surface temperature and the El Niño-Southern Oscillation, and a candidate mechanism in global photosynthesis. *Atmos*. *Chem*. *Phys*. **15**, 11571–11592 (2015).

