# Supplementary material for: Recent pause in the growth rate of atmospheric CO2 due to enhanced terrestrial carbon uptake
Source: Nat Commun. 2016 Nov 8;7:13428. doi: 10.1038/ncomms13428 (PMC5105171; doi:10.1038/ncomms13428)
Supplement: Supplementary Information — Supplementary Figures 1-12 and Supplementary Tables 1-3 Supplementary References. [file ncomms13428-s1.pdf]

Supplementary Figures

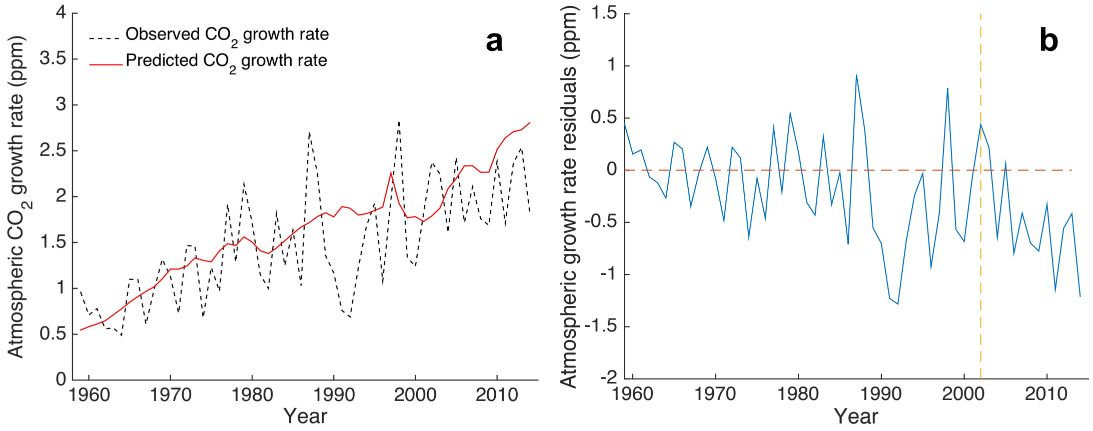

Supplementary Figure 1 | Divergence of the atmospheric CO_2 growth rate from a linear model of atmospheric CO_2. (*a*) Observed (black dashed line) and modeled (solid red line) growth rate, and (***b***) residuals between the observed and predicted atmospheric CO_2 growth rate from the linear model. A clear divergence is visible during this century, which is significant from 2002 (at $p < 0.05$, vertical orange dashed line on panel ***b***), indicating a change in the efficiency of global CO_2 sinks.

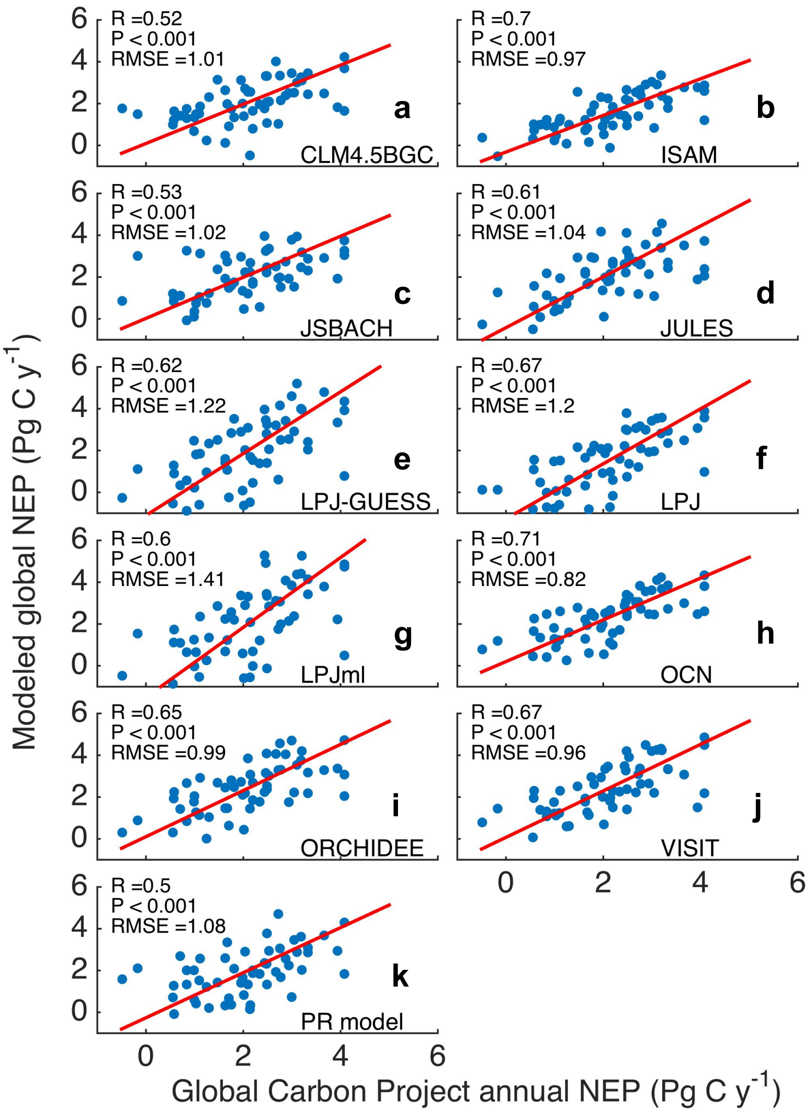

Supplementary Figure 2 | Assessment of modeled interannual variability in net ecosystem production. Estimates of net ecosystem production between 1959 and 2014 from ten dynamic global vegetation models (***a* - *j***) used in the Global Carbon Project, compared to estimated net ecosystem production using the Global Carbon Project net balance residual approach. Results from the diagnostic model (PR model) used in this study are also provided (***k***) for comparison. Red lines represent reduced major axis regressions. Model names and details can be found in Supplementary Table 1.

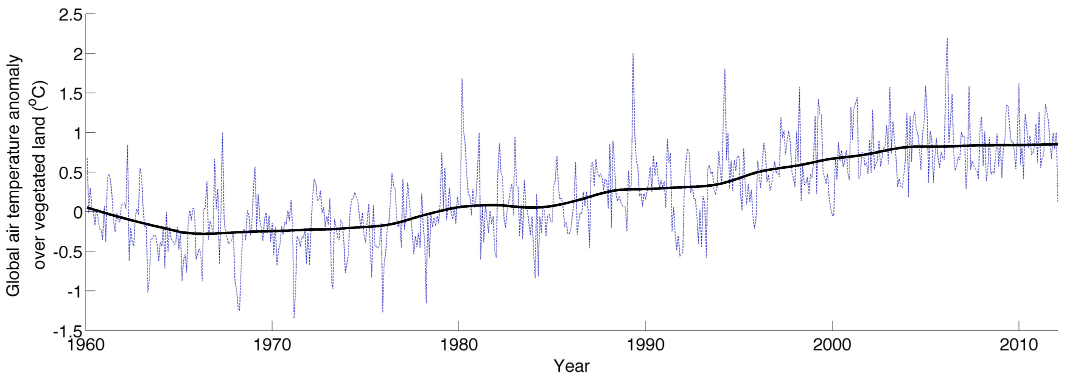

Supplementary Figure 3 | Long term warming over vegetated land. Monthly global air temperature anomalies over land from 1960 to 2012 (dashed blue line) calculated relative to the mean over that period. Temperature data are available from the Climate Research Unit at East Anglia University (CRU TS3.21)[1]. The black line represents the underlying decadal variability estimated using singular spectrum analysis (see methods).

(a)

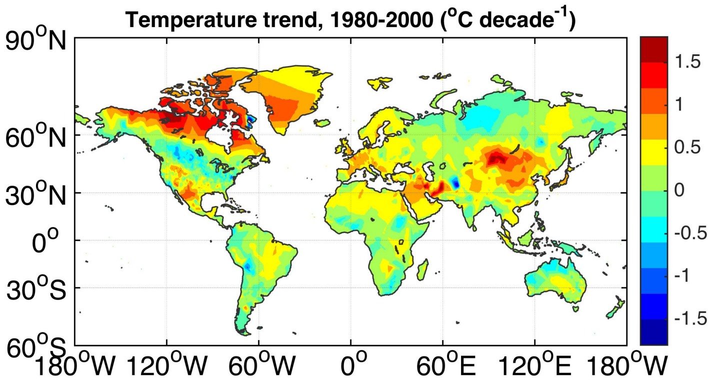

(b)

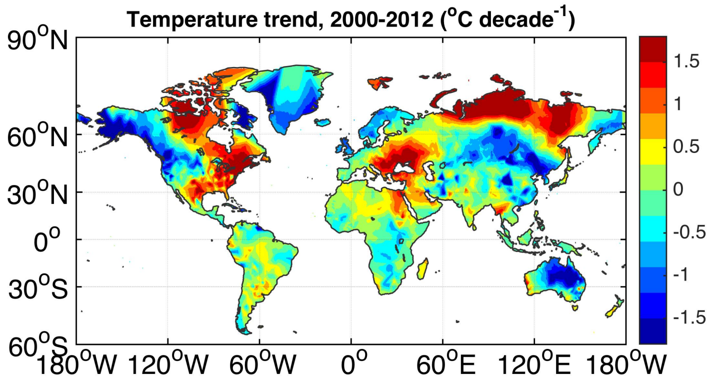

(c)

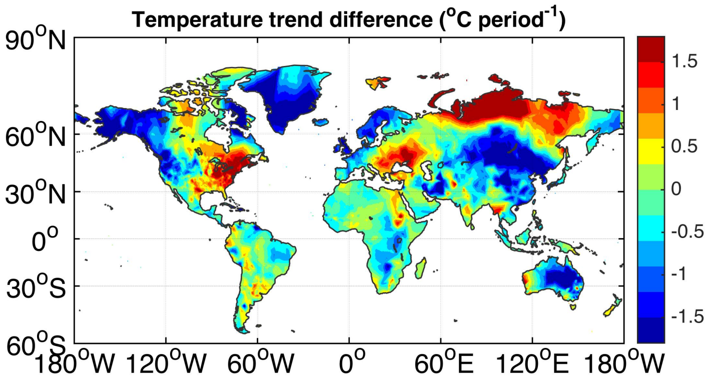

Supplementary Figure 4 | Spatial distribution of warming over vegetated land. (a, b) Decadal trends in air temperature (a: for 1980-2000, b: for 2000-2012) derived from monthly de-seasonalized surface temperature data using the Sen slope from Kendall's Tau-b method. **(c)** The difference in temperature trends between the two periods examined. Negative numbers indicate reduced warming, positive numbers indicate increased warming. Temperature data are available from the Climate Research Unit at East Anglia University (CRU TS3.21[1]).

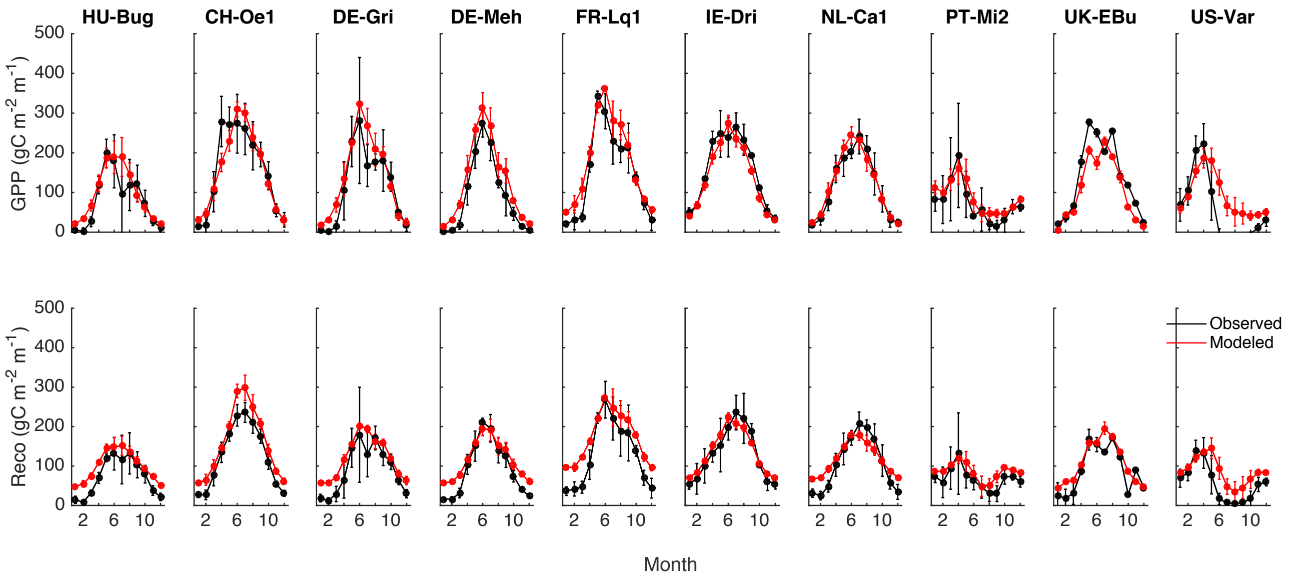

Supplementary Figure 5 | PR model validation at grassland sites: site-specific mean monthly comparisons of the diagnostic PR model projections of *GPP* and R_{eco} (red lines, dots) with observations (black lines, dots) for select (10 out of 21 available) grassland sites in the FLUXNET La Thuile Fair Use database. Error bars represent one standard deviation of monthly values. Site details are given in Supplementary Table 3.

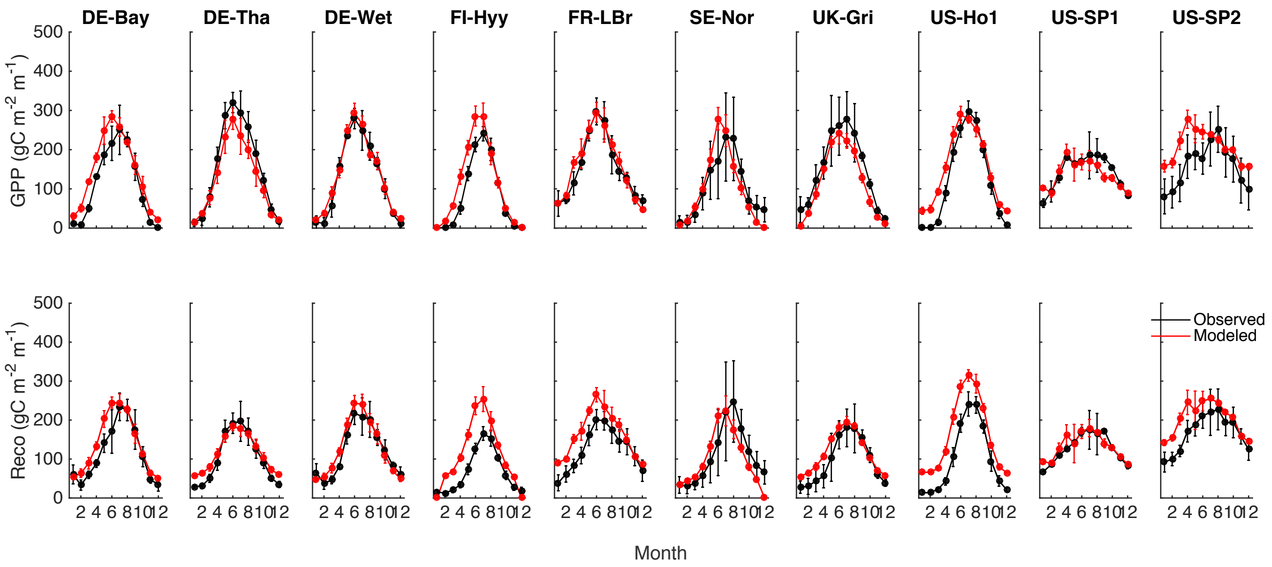

Supplementary Figure 6 | Evergreen needleleaf forests: site-specific mean monthly comparisons of the diagnostic PR model projections of *GPP* and R_{eco} (red lines, dots) with observations (black lines, dots) for select (10 out of 31 available) evergreen needleleaf forest sites in the FLUXNET La Thuile Fair Use database. Error bars represent one standard deviation of monthly values. Site details are given in Supplementary Table 3.

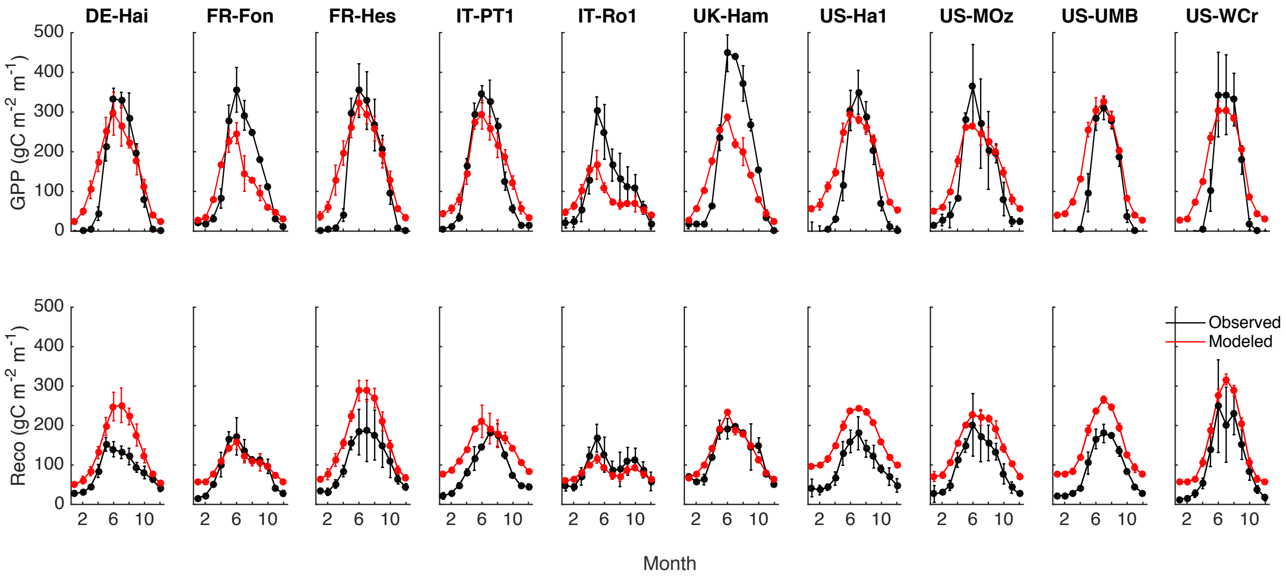

Supplementary Figure 7 | Deciduous broadleaf forests: site-specific mean monthly comparisons of the diagnostic PR model projections of *GPP* and R_{eco} (red lines, dots) with observations (black lines, dots) for select (10 out of 19 available) deciduous broadleaf forest sites in the FLUXNET La Thuile Fair Use database. Error bars represent one standard deviation of monthly values. Site details are given in Supplementary Table 3.

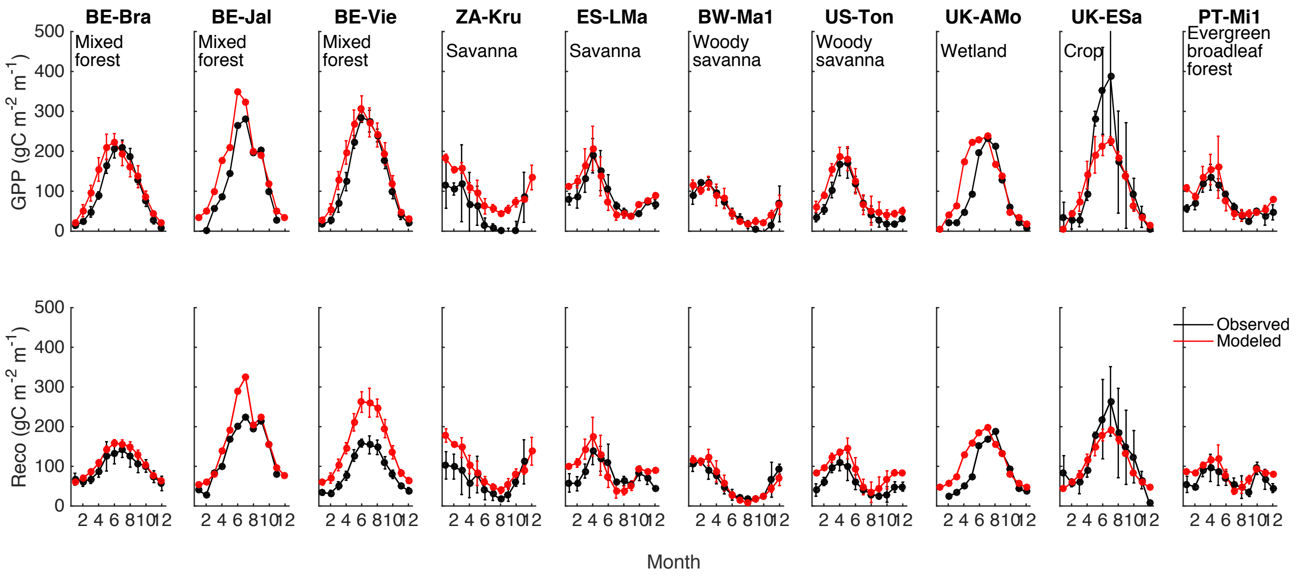

Supplementary Figure 8 | Site-specific mean monthly comparisons of the diagnostic PR model projections of *GPP* and R_{eco} (red lines, dots) with observations (black lines, dots) for 10 representative sites for mixed forests, savannahs, woody savannahs, a crop, wetland and evergreen broadleaf forest site in the FLUXNET La Thuile Fair Use database. Error bars represent one standard deviation of monthly values. Site details are given in Supplementary Table 3.

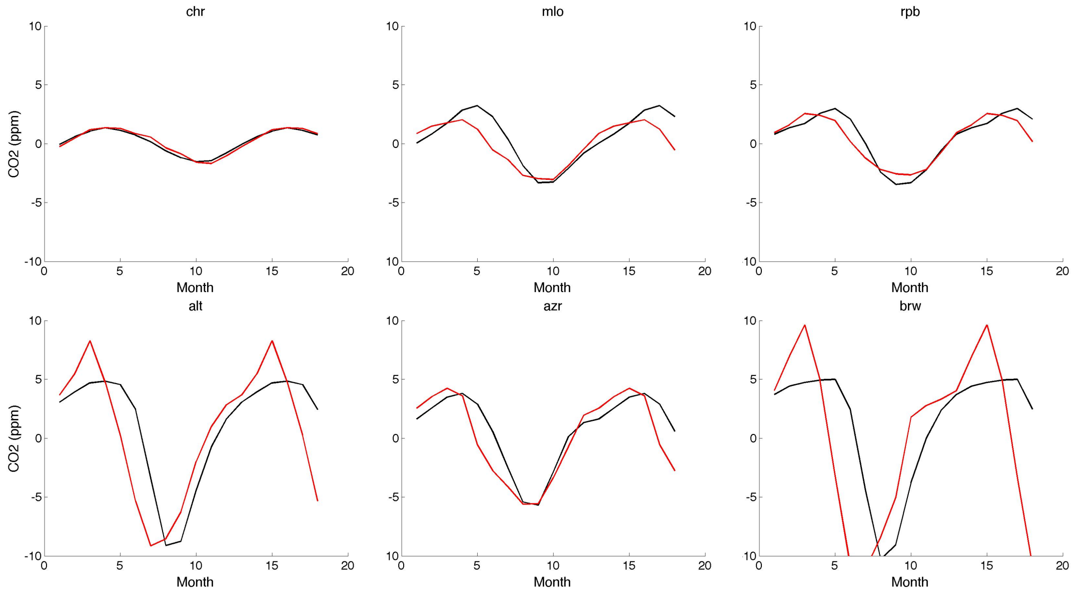

Supplementary Figure 9 | Comparison to seasonal cycle of NOAA station data

At each station, the modeled atmospheric CO_2 concentrations (red) captures both the timing and the amplitude of the seasonal variability in observed atmospheric CO_2 concentrations (black). Station names represented are: CHR: Christmas Island (2°N 157°W); MLO: Mauna Loa (20°N 156°W); RPB: Ragged Point (13°N 59°W); ALT: Alert (82°N 63°W); AZR: Azores (39°N 27°W); BRW: Point Barrow (71°N 157°W).

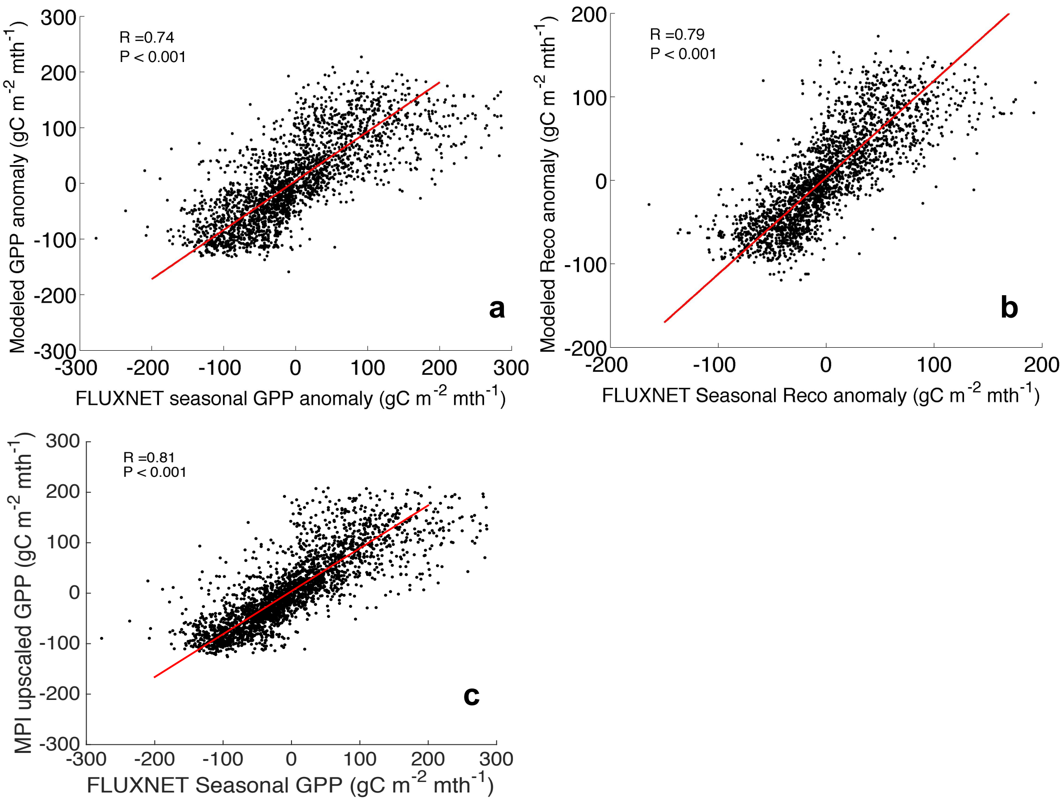

Supplementary Figure 10 | Comparison of model estimates of gross primary production and ecosystem respiration with observations from 153 globally distributed sites in the FLUXNET La Thuile dataset

Black dots represent monthly values of gross primary production (GPP, ***a***, ***c***) and ecosystem respiration (R_{eco}, ***b***), from each FLUXNET site, normalized on a site basis relative to the mean monthly value over all years at each site. Monthly values are compared for the diagnostic photosynthesis-respiration (PR) model (***a***, ***b***) and the Max-Planck Research Institute (MPI) upscaled estimates of GPP (***c***). Red lines indicate a reduced major-axis (type II) regression.

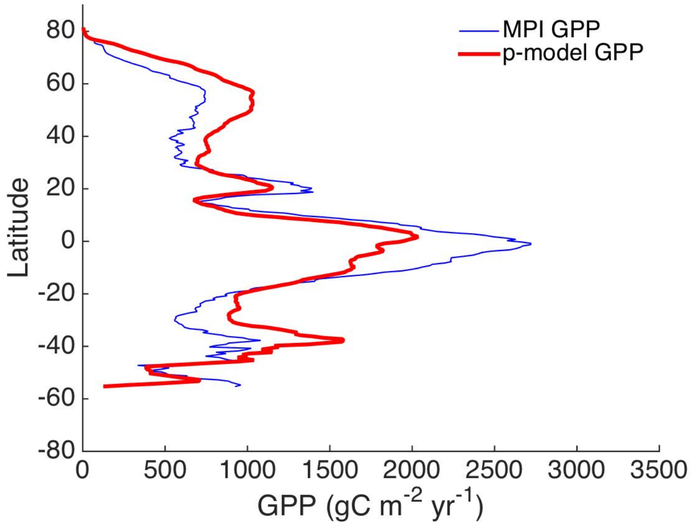

Supplementary Figure 11 | Latitudinal comparison to an empirical upscaling product. Comparing modeled gross primary production (GPP) from the photosynthetic module (the P model, red line) of the diagnostic light use efficiency model (PR model) and the MPI empirical upscaling approach (blue line).

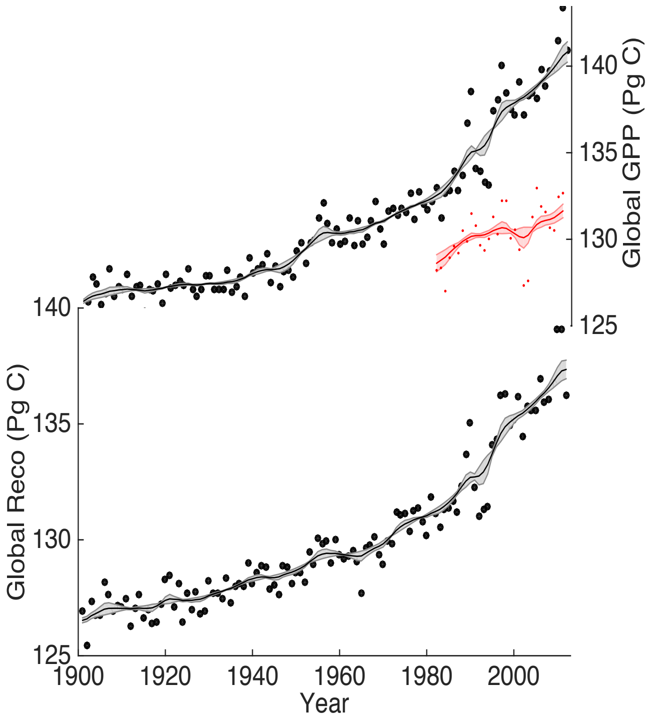

Supplementary Figure 12 | Diagnostic model projections of the long-term change in global gross ecosystem production and respiration. Diagnostic PR model predictions of global gross ecosystem production (*GPP*), and ecosystem respiration (R_{eco}) from 1901 to 2012. Black dots represent annual values, and grey lines represent long term temporal dynamics estimated using singular spectrum analysis. The red dots and shaded area represent global *GPP* estimates from up-scaled eddy-covariance flux measurements (The MPI *GPP* product).

Supplementary Tables

Supplementary Table 1 | The dynamic global vegetation models used. Note that results shown in Fig. 4 and Fig. 5 for the 'Climate only' simulations represent the five models: HYLAND, LPJ, ORCHIDEE, SDGVM, TRIFFID.

Global Carbon Budget DGVMs	
**Model**	**Reference**
CLM4.5BGC	Oleson et al (2013)[2]
ISAM	Jain et al. (2013)[3]
JSBACH	Reick et al. (2013)[4]
JULES	Clark et al. (2011)[5]
LPJ-GUESS	Krinner et al. (2005)[6]
LPJ	Smith et al. (2003)[7]
LPJml	Sitch et al. (2003)[7]
OCN	Bondeau et al. (2007)[8]
ORCHIDEE	Zaehle et al. (2010)[9]
VISIT	Kato et al. (2013)[10]

TRENDY models	
**Model**	**Reference**
LPJ Guess	Krinner et al. (2005)[6]
LPJ	Smith et al. (2003)[7]
OCN	Bondeau et al. (2007)[8]
CLM4CN	Bondeau et al. (2007)[8]
HYLAND	Levy et al. (2004)[11]
SDGVM	Cramer et al. (2001)[12]
TRIFFID	Clark et al. (2011)[5]
VEGAS 2.1	Zeng et al. (2005)[13]

Supplementary Table 2 | FLUXNET sites used for site-specific comparisons.

Site Code	Name	Latitude	Longitude	Reference
**Deciduous Broadleaf forest (DBF)**				
DE-Hai	Hainich, Germany	51.0793	10.452	A. Knohl *et al.*, (2003)[14]
FR-Fon	Fontainebleau, France	48.4763	2.7802	-
FR-Hes	Hesse, France	48.6742	7.0646	A. Granier, *et al.*, (2000)[15]
IT-PT1	Zerbolo-Parco, Italy	45.2009	9.061	M. Migliavacca, *et al.*, (2009)[16]
IT-Ro1	Roccarespampani, Italy	42.4081	11.93	A. Rey, *et al.*, (2002)[17]
UK-Ham	Hampshire, UK	51.1535	-0.8583	-
US-Ha1	Harvard Forest, USA	42.5378	-72.1715	S. Urbanski, *et al.*, (2007)[18]
US-MOz	Missouri Ozark, USA	38.7441	-92.2	L. H. Gu, *et al.*, (2006)[19]
US-UMB	U. Mich. Biological Station, USA	45.5598	-84.7138	C. M. Gough et al., (2008)[20]
US-WCr	Willow Creek, USA	45.8059	-90.0799	B. D. Cook, *et al.*, (2004)[21]
**Evergreen Needleleaf Forest (ENF)**				
DE-Bay	Bayreuth-Waldstein, Germany	50.1419	11.8669	K. Staudt, and T. Foken (2007)[22]
DE-Tha	Tharandt, Germany	50.9636	13.5669	T. Grunwald *et al.*, (2007)[23]

DE-Wet	Wetzstein, Germany	50.4535	11.4575	C. Rebmann, *et al.*, (2010)[24]
FI-Hyy	Hyytiala, Finland	61.8474	24.2948	T. Suni, *et al.*, (2003)[25]
FR-LBr	Le Bray, France	44.7171	-0.7693	P. Berbigier *et al.*, (2001)[26]
SE-Nor	Norunda, Sweden	60.0865	17.4795	F. Lagergren, *et al.*, (2008) [27]
UK-Gri	Griffin-Aberfeldy, UK	56.6072	-3.7981	C. Rebmann, *et al.*, (2005) [28]
US-Ho1	Howland, USA	45.2041	-68.7402	D. Hollinger, *et al.*, (2004)[29]
US-SP1	Slashpine-Austin, USA	29.7381	-82.2188	T. L. Powell, *et al.*, (2008)[30]
US-SP2	Slashpine-Mize, USA	29.7648	-82.2448	K. L. Clark *et al.*, (2004)[31]
**Grassland (GRA)**				
HU-Bug	Bugacpuszta, Hungary	46.6911	19.6013	Z. Nagy, *et al.*, (2007)[32]
CH-Oe1	Oensingen, Switzerland	47.2856	7.7321	C. Ammann *et al.*, (2007)[33]
DE-Gri	Grillenburg, Germany	50.9495	13.5125	T. Gilmanov, *et al.*, (2007)[34]
DE-Meh	Mehrstedt, Germany	51.2753	10.6555	A. Don *et al.*, (2009)[35]
FR-Lq1	Laqueuille, France	45.6441	2.737	T. Gilmanov, *et al.*, (2007)[34]
IE-Dri	Dripsey, Ireland	51.9867	-8.7518	-
NL-Ca1	Cabauw, Netherlands	51.971	4.927	-
PT-Mi2	Mitra, Portugal	38.5407	-8.0004	J. S. Pereira, *et al.*, (2007)[36]
UK-EBu	Easter Bush, UK	55.866	-3.2058	-
US-Var	Vaira Ranch, USA	38.4133	-120.9507	L. Xu *et al.*, (2004)[37]

Miscellaneous PFTs

BE-Bra	Brasschaat, Belguim	51.3092	4.5206	A. Carrara *et al.*, (2003)[38]
BE-Jal	Jalhay, Belguim	50.5639	6.0733	-
BE-Vie	Vielsalm, Belguim	50.3055	5.9968	M. Aubinet, *et al.*, (2001)[39]
ZA-Kru	Skukuza- Kruger National Park, South Africa	-25.0197	31.4969	Scholes *et al.*, (2001)[40]
ES-LMa	Las Majadas del Tietar, Spain	39.9415	-5.7734	-
BW-Ma1	Maun-Mopane, Botswana	-19.9155	23.5605	E. M. Veenendaal, *et al.*, (2004)[41]
US-Ton	Tonzi Ranch, USA	38.4316	-120.966	S. Y. Ma *et al.*, (2007)[42]
UK-AMo	Auchencorth, UK	55.7917	-3.2389	-
UK-ESa	East Saltoun, UK	55.9069	-2.8586	-
PT-Mi1	Mitra, Portugal	38.5407	-8.0004	J. S. Pereira, *et al.*, (2007)[36]

Supplementary Table 3 | The declining sensitivity of photosynthesis to CO_2.

C_a	β_{CO2}
340 ppm during 1980	43%
**400 ppm, 2015 level**	**37%**
425 ppm	35%
510 ppm, 1.5 times the level in 1980	29%
595 ppm	25%
680 ppm, double the level in 1980	22%
**800 ppm, double the 2015 level**	**19%**

Supplementary References*:*

1. Harris, I., Jones, P. D., Osborn, T. J. & Lister, D. H. Updated high-resolution grids of monthly climatic observations - the CRU TS3.10 Dataset. *Int. J. Climatol.* **34,** 623–642 (2014).
2. Oleson, K. W. *et al. Technical description of version 4.0 of the Community Land Model (CLM). NCAR Technical Note* (2013). doi:10.5065/D6RR1W7M
3. Jain, A. K., Meiyappan, P., Song, Y. & House, J. I. CO2 emissions from land-use change affected more by nitrogen cycle, than by the choice of land-cover data. *Glob. Chang. Biol.* **19,** 2893–2906 (2013).
4. Reick, C. H., Raddatz, T., Brovkin, V. & Gayler, V. Representation of natural and anthropogenic land cover change in MPI-ESM. *J. Adv. Model. Earth Syst.* **5,** 459–482 (2013).
5. Clark, D. B. *et al.* The Joint UK Land Environment Simulator (JULES), model description – Part 2: Carbon fluxes and vegetation dynamics. *Geosci. Model Dev.* **4,** 701–722 (2011).
6. Krinner, G. *et al.* A dynamic global vegetation model for studies of the coupled atmosphere-biosphere system. *Global Biogeochem. Cycles* **19,** GB1015 (2005).
7. Sitch, S. *et al.* Evaluation of ecosystem dynamics, plant geography and terrestrial carbon cycling in the LPJ dynamic global vegetation model. *Glob. Chang. Biol.* **9,** 161–185 (2003).
8. Bondeau, A. *et al.* Modelling the role of agriculture for the 20th century global terrestrial carbon balance. *Glob. Chang. Biol.* **13,** 679–706 (2007).
9. Zaehle, S. & Friend, A. D. Carbon and nitrogen cycle dynamics in the O-CN land surface model: 1. Model description, site-scale evaluation, and sensitivity to parameter estimates. *Global Biogeochem. Cycles* **24,** 1–13 (2010).
10. Kato, E., Kinoshita, T., Ito, A., Kawamiya, M. & Yamagata, Y. Evaluation of spatially explicit emission scenario of land-use change and biomass burning using a process-based biogeochemical model. *J. Land Use Sci.* **8,** 104–122 (2013).
11. Levy, P. E., Cannell, M. G. R. & Friend, A. D. Modelling the impact of future changes in climate, CO2 concentration and land use on natural ecosystems and the terrestrial carbon sink. *Glob. Environ. Chang.* **14,** 21–30 (2004).
12. Cramer, W. *et al.* Global response of terrestrial ecosystem structure and

function to CO 2 and climate change: results from six dynamic global vegetation models. *Glob. Chang. Biol.* **7,** 357–373 (2001).

13. Zeng, N., Qian, H., Roedenbeck, C. & Heimann, M. Impact of 1998-2002 midlatitude drought and warming on terrestrial ecosystem and the global carbon cycle. *Geophys. Res. Lett.* **32,** n/a–n/a (2005).
14. Knohl, A., Schulze, E. D., Kolle, O. & Buchmann, N. Large carbon uptake by an unmanaged 250-year-old deciduous forest in Central Germany. *Agric. For. Meteorol.* **118,** 151–167 (2003).
15. Granier, A. *et al.* The carbon balance of a young Beech forest. *Funct. Ecol.* **14,** 312–325 (2000).
16. Migliavacca, M. *et al.* Modeling gross primary production of agro-forestry ecosystems by assimilation of satellite-derived information in a process-based model. *Sensors* **9,** 922–942 (2009).
17. Rey, A. *et al.* Summary for Policymakers. *Clim. Chang. 2013 - Phys. Sci. Basis* **33,** 1–30 (2002).
18. Urbanski, S. *et al.* Factors controlling CO 2 exchange on timescales from hourly to decadal at Harvard Forest. *J. Geophys. Res.* **112,** 1–25 (2007).
19. Gu, L. *et al.* Direct and indirect effects of atmospheric conditions and soil moisture on surface energy partitioning revealed by a prolonged drought at a temperate forest site. *J. Geophys. Res. Atmos.* **111,** 1–13 (2006).
20. Gough, C. M., Vogel, C. S., Schmid, H. P., Su, H. B. & Curtis, P. S. Multi-year convergence of biometric and meteorological estimates of forest carbon storage. *Agric. For. Meteorol.* **148,** 158–170 (2008).
21. Cook, B. D. *et al.* Carbon exchange and venting anomalies in an upland deciduous forest in northern Wisconsin, USA. *Agric. For. Meteorol.* **126,** 271–295 (2004).
22. Staudt, K. & Foken, T. *Documentation of reference data for the experimental areas of the Bayreuth Centre for Ecology and Environmental Research (BayCEER) at the Waldstein site.* (2007).
23. Grünwald, T. & Bernhofer, C. A decade of carbon, water and energy flux measurements of an old spruce forest at the Anchor Station Tharandt. *Tellus, Ser. B Chem. Phys. Meteorol.* **59,** 387–396 (2007).
24. Rebmann, C. *et al.* Treatment and assessment of the CO2-exchange at a complex forest site in Thuringia, Germany. *Agric. For. Meteorol.* **150,** 684–691

(2010).

25. Suni, T., Rinne, J., Reissell, A. & Altimir, N. Long-term measurements of surface fluxes above a Scots pine forest in Hyytiälä, Finland, 1996-2001. *Boreal Environ. Res.* **8,** 287–301 (2003).

26. Berbigier, P., Bonnefond, J.-M. & Mellmann, P. CO2 and water vapour fluxes for 2 years above Euroflux forest site. *Agric. For. Meteorol.* **108,** 183–197 (2001).

27. Lagergren, F. *et al.* Biophysical controls on CO2 fluxes of three Northern forests based on long-term eddy covariance data. *Tellus, Ser. B Chem. Phys. Meteorol.* **60 B,** 143–152 (2008).

28. Rebmann, C. *et al.* Quality analysis applied on eddy covariance measurements at complex forest sites using footprint modelling. *Theor. Appl. Climatol.* **80,** 121–141 (2005).

29. Hollinger, D. Y. *et al.* Spatial and temporal variability in forest-atmosphere CO2 exchange. *Glob. Chang. Biol.* **10,** 1689–1706 (2004).

30. Powell, T. L. *et al.* Carbon exchange of a mature, naturally regenerated pine forest in north Florida. *Glob. Chang. Biol.* **14,** 2523–2538 (2008).

31. Clark, K. K. L., Gholz, H. L. H. & Castro, M. S. M. Carbon dynamics along a chronosequence of slash pine plantations in north Florida. *Ecol. Appl.* **14,** 1154–1171 (2004).

32. Nagy, Z. *et al.* The carbon budget of semi-arid grassland in a wet and a dry year in Hungary. *Agric. Ecosyst. Environ.* **121,** 21–29 (2007).

33. Ammann, C., Flechard, C. R., Leifeld, J., Neftel, A. & Fuhrer, J. The carbon budget of newly established temperate grassland depends on management intensity. *Agric. Ecosyst. Environ.* **121,** 5–20 (2007).

34. Gilmanov, T. G. *et al.* Partitioning European grassland net ecosystem CO2 exchange into gross primary productivity and ecosystem respiration using light response function analysis. *Agric. Ecosyst. Environ.* **121,** 93–120 (2007).

35. Don, A., Rebmann, C., Kolle, O., Scherer-Lorenzen, M. & Schulze, E. D. Impact of afforestation-associated management changes on the carbon balance of grassland. *Glob. Chang. Biol.* **15,** 1990–2002 (2009).

36. Pereira, J. S. *et al.* Net ecosystem carbon exchange in three contrasting Mediterranean ecosystems – the effect of drought. *Biogeosciences* **4,** 791–802 (2007).

37. Xu, L. & Baldocchi, D. D. Seasonal variation in carbon dioxide exchange over a Mediterranean annual grassland in California. *Agric. For. Meteorol.* **123,** 79–96 (2004).
38. Carrara, A. *et al.* Net ecosystem CO2 exchange of mixed forest in Belgium over 5 years. *Agric. For. Meteorol.* **119,** 209–227 (2003).
39. Aubinet, M. *et al.* Long term carbon dioxide exchange above a mixed forest in the Belgian Ardennes. *Agric. For. Meteorol.* **108,** 293–315 (2001).
40. Scholes, R. J. *et al.* The environment and vegetation of the flux measurement site near Skukuza, Kruger National Park. *Koedoe* **44,** 73–84 (2001).
41. Veenendaal, E. M., Kolle, O. & Lloyd, J. Seasonal variation in energy fluxes and carbon dioxide exchange for a broad-leaved semi-arid savanna (Mopane woodland) in Southern Africa. *Glob. Chang. Biol.* **10,** 318–328 (2004).
42. Ma, S., Baldocchi, D., Xu, L. & Hehn, T. Inter-annual variability in carbon dioxide exchange of an oak/grass savanna and open grassland in California. *Agric. For. Meteorol.* **147,** 157–171 (2007).
